# Structural basis for nuclear import of bat adeno-associated virus capsid protein

**DOI:** 10.1099/jgv.0.001960

**Published:** 2024-03-05

**Authors:** Mikayla Hoad, Justin A. Roby, Jade K. Forwood

**Affiliations:** 1 School of Dentistry and Medical Sciences, Charles Sturt University, Wagga Wagga, NSW, 2678, Australia; 2 Gulbali Institute, Charles Sturt University, Wagga Wagga, NSW, 2678, Australia

**Keywords:** nuclear import, adeno-associated virus, importin, karyopherin

## Abstract

Adeno-associated viruses (AAV) are one of the world’s most promising gene therapy vectors and as a result, are one of the most intensively studied viral vectors. Despite a wealth of research into these vectors, the precise characterisation of AAVs to translocate into the host cell nucleus remains unclear. Recently we identified the nuclear localization signals of an AAV porcine strain and determined its mechanism of binding to host importin proteins. To expand our understanding of diverse AAV import mechanisms we sought to determine the mechanism in which the Cap protein from a bat-infecting AAV can interact with transport receptor importins for translocation into the nucleus. Using a high-resolution crystal structure and quantitative assays, we were able to not only determine the exact region and residues of the N-terminal domain of the Cap protein which constitute the functional NLS for binding with the importin alpha two protein, but also reveal the differences in binding affinity across the importin-alpha isoforms. Collectively our results allow for a detailed molecular view of the way AAV Cap proteins interact with host proteins for localization into the cell nucleus.

## Introduction

Adeno-associated viruses (AAVs) are classified within the genus *Dependoparvovirus* and the family *Parvoviridae* [1,2]. They are small single-stranded DNA viruses, with a linear genome (~4.7 kilobases) that encodes for three genes, *Rep*, *Cap* and *Aap*, flanked by inverted terminal repeats [3]. There are at least 13 different recognized serotypes of AAV isolated from human and non-human primate tissues [4,5] with over ~80 % of the human population seropositive for at least one form of AAV [6]. Known for infecting vertebrates but retaining a minimal capacity to invoke the immune response [7], an inability to manifest any phenotypic diseases [8], and an incapacity to replicate without the co-infection of the host with another virus [8,10]; AAVs have become established as strong candidates for use as gene therapy vectors. Moreover, AAVs can exhibit specific tissue tropism [11,12], and the ease with which they can be adapted to different tropisms through genetic engineering [12,16] makes these viruses the most extensively researched therapeutic viral vector for gene therapy.

There are currently over 130 clinical trials [17] which use AAV as a therapeutic vector, with three AAV viral vectors approved and marketed as drugs: Glybera (lipoprotein lipase deficiency), Luxturnan (Leber congenital amaurosis), and Zolgensma (spinal muscular atrophy) [18]. AAV exemplifies a robust gene therapy vector, although varying levels of efficiency have been observed in different studies [19,20]. The range of diseases that AAV vectors can be utilised to treat is broad, and could include cancer, neurological and central nervous system diseases such as Parkinson’s disease, illnesses affecting vision, blood diseases such as haemophilia, and muscular diseases [21,25]. However, AAVs implementation as gene therapy vectors does present challenges. Due to the high prevalence of human exposure to at least one AAV serotype, anamnestic antibodies in patients have the potential to neutralize therapeutic particles [26,28]. Thus, AAV isolates from animals other than humans (for which the likelihood of patient exposure is negligible) have been explored as alternative vectors [29,31]. Indeed, a recent publication has demonstrated that AAV capsids derived from a bat-infecting viral isolate are able to escape neutralization by human antibodies [32].

Considering how intensively studied AAVs are as gene therapy vectors, there is a large gap in understanding the precise mechanisms by which AAV vectors translocate into the host nucleus. The nuclear localization of AAV viral vectors is crucial for their utilisation as a gene therapy tool [33,34], as correct translocation from the cytoplasm to the nucleus is required for therapeutic transgene DNA delivery. A thorough understanding of the mechanisms through which AAVs translocate to the nucleus (including the molecular mechanisms which AAV uses to interact with host nuclear import proteins) will facilitate a potential basis for improving transduction of targeted cells, and thus the overall efficiency of AAV viral vectors.

The classical nuclear import pathway comprises a cargo protein within the cytoplasm binding to a host transport adaptor protein, importin-alpha (IMPα), via a series of specific, basic amino acid residues known as a nuclear localization signal (NLS) [35]. These motifs may be either a monopartite NLS (binding to only one cargo-interaction site on IMPα) or a bipartite NLS (binding to both the major and minor cargo-interaction sites). Once host IMPα binds to the NLS of the cargo protein [36], another host nuclear transport protein, importin-beta (IMPβ), binds to a region of IMPα (the IMPβ-binding domain or IBB) and facilitates movement of the entire trimer through the nuclear pore complex and into the nuclear lumen of the cell [37]. On some occasions [38,39] cargo proteins can bind directly to IMPβ proteins for movement into the nucleus, excluding the adaptor IMPα protein. Once the cargo:IMPα:IMPβ, or cargo:IMPβ complex has travelled through the nuclear pore, Ran-GTP dissociates the complex and IMPα and IMPβ proteins are recycled back into the cell cytoplasm to continue facilitating the nuclear import of cargo proteins [40,41]. It has been shown [42,45] that AAV capsid proteins can interact with host importin proteins to enter the nucleus.

**Fig. 1.**
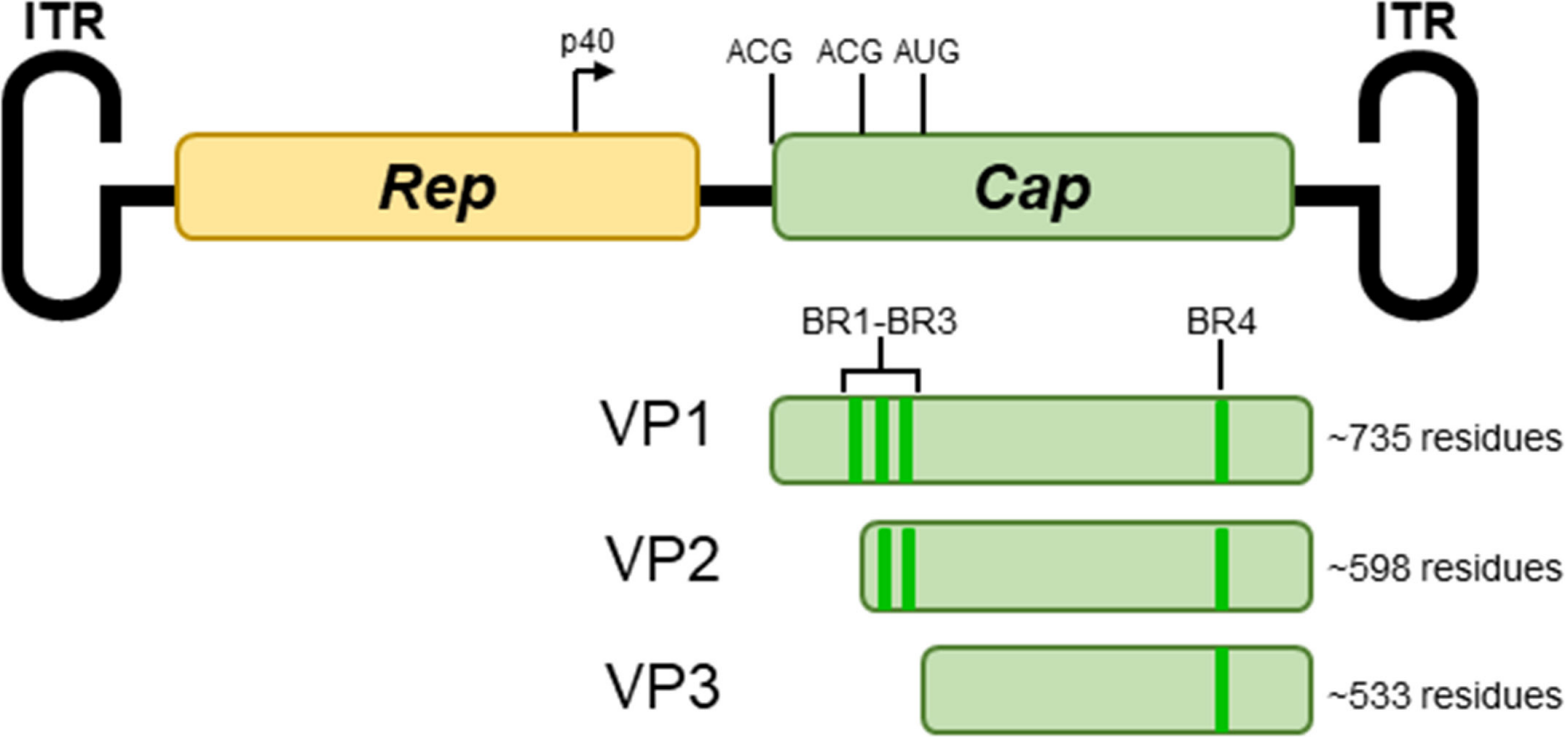
AAV genome and *Cap* gene products. Schematic overview of the AAV genome, including labelled ITRs, *Rep* gene, *Cap* gene, p40 promotor, and each VP start codon. Proteinaceous *Cap* gene products VP1-3 are shown with labelled BRs and an approximate size of each protein (varying slightly by AAV isolate).

AAV capsids are composed of three virion proteins (VP), translated from two mRNA splice variants [46,49]. VP1 (the largest of the VP proteins) contains the entirety of the open reading frame of the *Cap* gene (the entirety of protein sequence coded on mRNA transcribed from the p40 promotor); whilst VP2 and VP3 are produced from alternate downstream start codons (Fig. 1). VP2 (the second largest VP protein) maintains the common C-terminal domain but only possesses ~65 residues of the N-terminal domain; whilst VP3 (the smallest VP protein) possesses no N-terminal domain, only the common C-terminus [50] (Fig. 1). These three VP proteins coalesce to form the capsid of AAV in a ratio of 1 : 1 : 10 (VP1:VP2:VP3) [51,52]. The capsid is formed with a total of 60 VP subunits assembled through interactions which allow for the formation of pores within the capsid connecting the external milieu to the inside of the virion shell [53]. Despite structures having been obtained of various AAV capsids, the N-terminal regions of VP1 and VP2 have thus far been unable to be visualised [51,54]. It is theorized that this could be due to the low amount of VP1 and VP2 within the capsid or that this region may have high flexibility [55]. Studies have postulated that weak protein density observed in the capsid interior are internalized VP1/VP2 N-terminal regions [54,56, 57]. Previous literature has also determined that the N-terminal regions of VP1 and VP2 become exposed externally to the virion in the cytoplasm prior to nuclear localization and for the facilitation of endosomal escape [34,58,62]. External presentation of the N-terminal domain of VP1 and VP2 has been hypothesised to also promote nuclear localization [42].

The AAV capsid protein, VP1, contains four motifs of positively charged residues termed ‘basic regions’ (BRs), and these (BR1, BR2, BR3, and BR4) are suspected of acting as NLSs for the transduction of AAV capsid into the nucleus [42]. Different isoforms of AAV VP possess different complements of BRs due to the difference in capsid protein length at the N-terminus [60]. VP1 possesses all four BRs, with BR1, BR2 and BR3 found in the N-terminal domain and BR4 localized to the common C-terminal domain [42]. VP2 only possesses BR2, BR3, and BR4, whilst VP3 only maintains the C-terminal domain BR4 [42]. It has been revealed that mutations of BR2 and BR3 hindered AAV2 VPs transduction by over ten-fold [42,44, 63] whilst mutations to BR4 resulted in defective capsid assembly but no inhibition of nuclear localization [42,63]. Co-immunoprecipitation assays showed mutations to BR2 and BR3 limited interactions with IMPβ, however, mutations to BR1 had little effect on IMPβ binding [44] despite mutation to BR1 negatively affecting the phenotype of nuclear import [64]. It was theorized by Nicholson and colleagues that different AAV serotypes utilized different import proteins for nuclear entry, and it is also possible that each of BR1–3 contributes to nuclear import differently in diverse serotypes of AAV [44]. This theory is supported by the fact that many AAV serotypes harbour similar, if not identical, BRs [42,44, 60] and yet have been shown by Nicholson and colleagues (and our preliminary studies) to vary in ability to bind importins.

We have revealed herein that the N-terminus of VP1, from an AAV isolate from bats (09YN), interacts with host IMPα2 via a bipartite NLS. Our high resolution (2.4 Å) structural evidence demonstrated that BR3 interacts with the IMPα2 major cargo-binding site and that a region just downstream of BR2 interacts with the minor cargo-binding site. Furthermore, we have revealed that AAV 09YN VP1 interactions with host importin proteins was selective, with all isoforms of IMPα able to bind the NLS whilst IMPβ remained unbound. This data has increased the overall understanding of how suspected NLS regions of AAV VP1 interact with host importin receptors for nuclear localization, further supporting data published previously [43,45].

## Methods

### Gene construct design

Previous studies have shown that a BR4 mutant AAV capsid was present in both the nucleus and cytoplasm to the same degree as WT, however did not form assembled capsids (thus BR4 is not involved in nuclear translocation, but is instead involved in virion assembly) [42], we focussed solely upon BRs 1–3 in this study. The VP1 protein sequence from a bat-derived AAV isolate (isolate 09YN) was sourced through GenBank (accession AWW87409.1). A gene fragment of the N-terminal domain encoding amino acid residues from AAV 09YN VP1 that encompass basic regions 1–3 (^112^ANALFQAKKRVLEPFGLVEGEPEPKKTPSVKRPHASPDSS SGVGKKGDQPARKRLDFG169; referred to herein as AAV 09YN Cap-BR) was codon optimized for expression in *E. coli*, synthesised, and cloned into the BamHI sites of the pGEX4T-1 vector by GenScript. An engineered N-terminal tobacco etch virus (TEV) protease site was also included within the synthetic construct for GST-tag cleavage and removal. Mouse IMPα2 (mImpα2DIBB; His tag, no TEV site) encoded by pET30a expression vector has been described previously [65].

Importin proteins used for fluorescence polarization experiments; human importin α1 (hImpα1DIBB; His tag, TEV site), α3 (hImpα3DIBB; His tag, TEV site), α5 (hImp5DIBB; His tag, TEV site), α7 (hImpα7DIBB; His tag, TEV site), β (hImpβ; HIS tag, TEV site), and mouse importin α2 (mImpα2DIBB; His tag, no TEV site) encoded by pET30a expression vector have all been described previously [65,67].

A synthetic FITC labelled AAV 09YN Cap-BR peptide (with Ahx linker on the N-terminus) (^117^QAKKRVLEPFGLVEGEPEPKKTPSVKRPHASPDSSSGVGKKGDQPAR KRLD^167^) was obtained from GenScript. Mutants of this FITC-tagged AAV 09YN Cap-BR peptide were designed based on structural data of IMPα2 binding to disrupt either the NLS major site, minor site, or both. These mutant peptides Cap-BR-mMINOR (^117^QAKKRVLEPFGLVEGEPEPKKTPSV*AA*P*A*ASPDSSSGVGKKGDQPARKRLD^167^), Cap-BR-mMAJOR (^117^QAKKRVLEPFGLVEGEPEPKKTPSVKRPHASPDSSSGVGKKGDQPA*AAA*LD^167^) and Cap-BR-mMINOR+mMAJOR (^117^QAKKRVLEPFGLVEGEPEPKKTPSV*AA*P*A*ASPDSSSGVGKKGDQPA*AAA*LD^167^) were obtained from GenScript.

### Recombinant expression and purification

Expression and co-purification of AAV 09YN Cap-BR and IMPα2 was performed as per co-purification described by Hoad and colleagues [43]. Fig. S1, available in the online version of this article shows the subsequent expression, and purification for complexing of AAV 09YN Cap-BR and IMPα2.

Overexpression of importin α1, α2, α3, α5, α7, and β was performed in *E. coli* pLysS cells using the auto-induction method [68]. Following 36 h growth at room temperature, bacterial cells were pelleted via centrifugation at 5232 ***g*** and then resuspended in His buffer A (50 mM phosphate buffer, 300 mM NaCl, 20 mM imidazole, pH 8) at a volume of 15 ml per 2 litres of culture and lysed via two freeze-thaw cycles. The whole cell extracts of all importins were then further lysed with 1 ml of 20 mg ml^−1^ lysozyme (Sigma-Aldrich, USA) and concurrently treated with 10 µl of 50 mg ml^−1^ DNase (Sigma-Aldrich, USA) per 50 ml of cell suspension at room temperature for 1 h on a tube roller. Soluble extract was subsequently harvested via centrifugation at 11269 ***g*** for 30 min followed by filtration of the supernatant (soluble extract) via a 0.45 µm low protein affinity filter. Soluble extracts were injected over a 5 ml HisTrap HP column (GE Healthcare, USA) and washed with twenty column volumes of His buffer A on an AKTApurifier FPLC (GE Healthcare, USA). The samples were eluted via increasing concentration gradient of imidazole (20 mM to 500 mM) (ChemSupply, Australia), and elution fractions were pooled. The proteins were further purified using size-exclusion chromatography on a HiLoad 26/60 Superdex 200 column (GE Healthcare, USA), pre-equilibrated in GST buffer A (50 mM Tris, 125 mM NaCl). Fractions corresponding to the correct molecular weight of importin proteins were collected and concentrated using an Amicon MWCO 10 kDa filter (Merck Millipore, USA), and subsequently aliquoted and stored at −80 °C. Samples were assessed for purity by SDS-PAGE (165 V for 30 min on a 4–12 % Bis-Tris plus gel [Life Technologies, USA]).

### Crystallization of AAV 09YN Cap-BR and IMPα2 complex

The hanging drop vapour diffusion method was utilized with the final crystallization condition containing a reservoir of 0.75 M Na citrate, 0.1 M HEPES pH 7.5, mixed in a 1 : 1 ratio with the protein solution, and crystalized at 22 °C. Rod-shaped crystals (170×12×10 µm) grew after 2 days of incubation. The crystals were cryoprotected in the reservoir solution incorporating 20 % glycerol, before being flash-cooled to −196 °C in liquid nitrogen.

### Data collection and structure determination

**Fig. 2.**
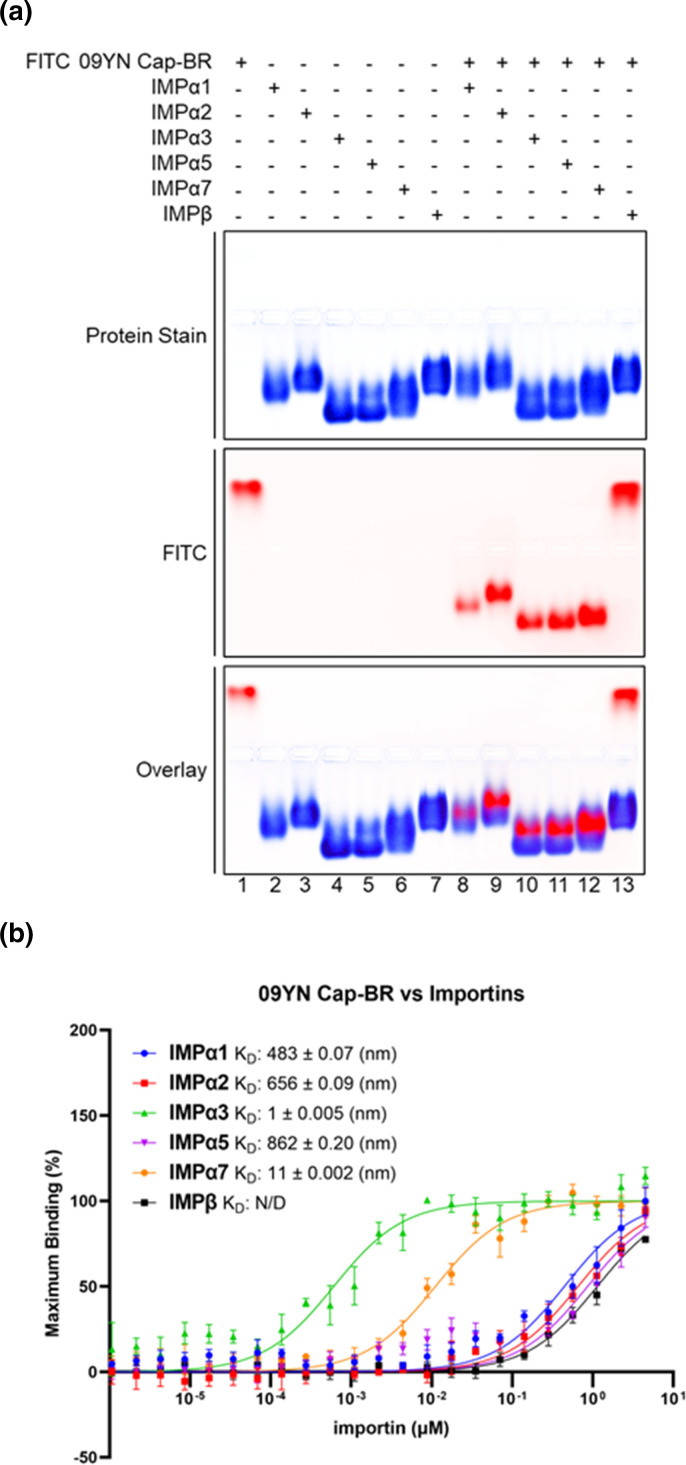
Crystal structure and binding interactions of AAV 09YN Cap-BR in complex with IMPα2. (**a**) Schematic overview of the AAV 09YN Cap protein and structure of AAV 09YN Cap-BR (green sticks) and IMPα2 (grey ribbons/transparent surface) complex resolved to 2.4 Å resolution. The sequence of AAV 09YN Cap-BR bound to IMPα2 shown in detail in the box. BRs 1, 2, and 3 are underlined. This structure has been deposited in the PDB and given the code: 8SG7. (**b**) Simplified representation of IMPα2 and AAV 09YN Cap-BR binding interactions. The AAV 09YN Cap-BR (green line) residues bound to IMPα2 (grey box) are indicated through complementary arrows. Salt bridges are indicated via underlined IMPα2 residues and non-underlined residues indicate hydrogen bonds indicated by the PDBsum server.

A single crystal was used to gather X-ray diffraction data at the Australian Synchrotron on the MX2 beamline [69]. The IMPα2 and AAV 09YN Cap-BR complex rod-shaped crystals (Fig. S1D) diffracted to 2.4 Å resolution (Table S1). The diffraction data was indexed and then merged in iMOSFLM [70], before being scaled and merged using Aimless (Table S1) [71,72]. Phasing was performed using molecular replacement in Phaser [73] with the Protein Data Bank (PDB) structure 4OIH as a search model. Model rebuilding and relative refinements were performed using Coot [74,76] and Phenix [77] programmes. Through rebuilding and refinement, the final model had a R_work_/R_free_ of 0.18/0.22. The finalized model (Fig. 2) consists of 426 residues of IMPα2 (72-497), 21 (139-149; 160-169) residues of AAV 09YN Cap-BR and 41 water molecules. The stereochemistry and other refinement statistics are presented in Table S1.

### Fluorescence polarization assay

Synthetic FITC-tagged AAV 09YN Cap-BR peptide was incubated (2 nm) with two-fold serially diluted IMPα concentrations (starting concentration 4.5 µM) across 23 wells to a complete volume of 200 µl per well with GST Buffer A (50 mM Tris, 125 mM NaCl). Fluorescence polarization (FP) measurements were recorded using a CLARIOstar Plus plate reader (BMG Labtech, Germany) in 96-well black Fluotrac microplates (Greiner Bio-One, Austria). Assays were performed as three independent experiments, each containing a negative control (no importin binding partner). Data from the three independent experiments was used to generate a binding curve using GraphPad Prism (Prism 9, Version 9.3.1).

### Electro-mobility shift assay (EMSA)

The FITC-tagged AAV 09YN Cap-BR peptide (10 µM) was mixed with 20 µM of each IMPα isoform and incubated in a total volume of 20 µl supplemented with 3 µl of 50 % glycerol and the remainder made up with GST Buffer A (50 mM Tris, 125 mM NaCl). Samples were run on a 1.5 % agarose TB gel (45 mM TRIZMA base, 45 mM boric acid, 1.5 % agarose, pH ~8.5) for 1.5 h at 70 V in TB running buffer (45 mM TRIZMA base, 45 mM boric acid, pH ~8.5). Images were recorded using a SYBR green filter of the GEL Doc BioRad Gel Doc imaging system (Bio-Rad Laboratories, USA). The gel was then stained using Coomassie stain (40 % ethanol, 10 % glacial acetic acid, 0.2 % Coomassie Brilliant Blue [R-250]) for 10 mins at room temperature and subsequently destained with 10 % ethanol and 10 % glacial acetic acid overnight prior to imaging with Gel Doc BioRad Gel Doc imaging system (Bio-Rad Laboratories, USA).

## Results

### Biochemical determination of AAV 09YN Cap-BR preference for importin alpha 3 and 7 isoforms

**Fig. 3.**
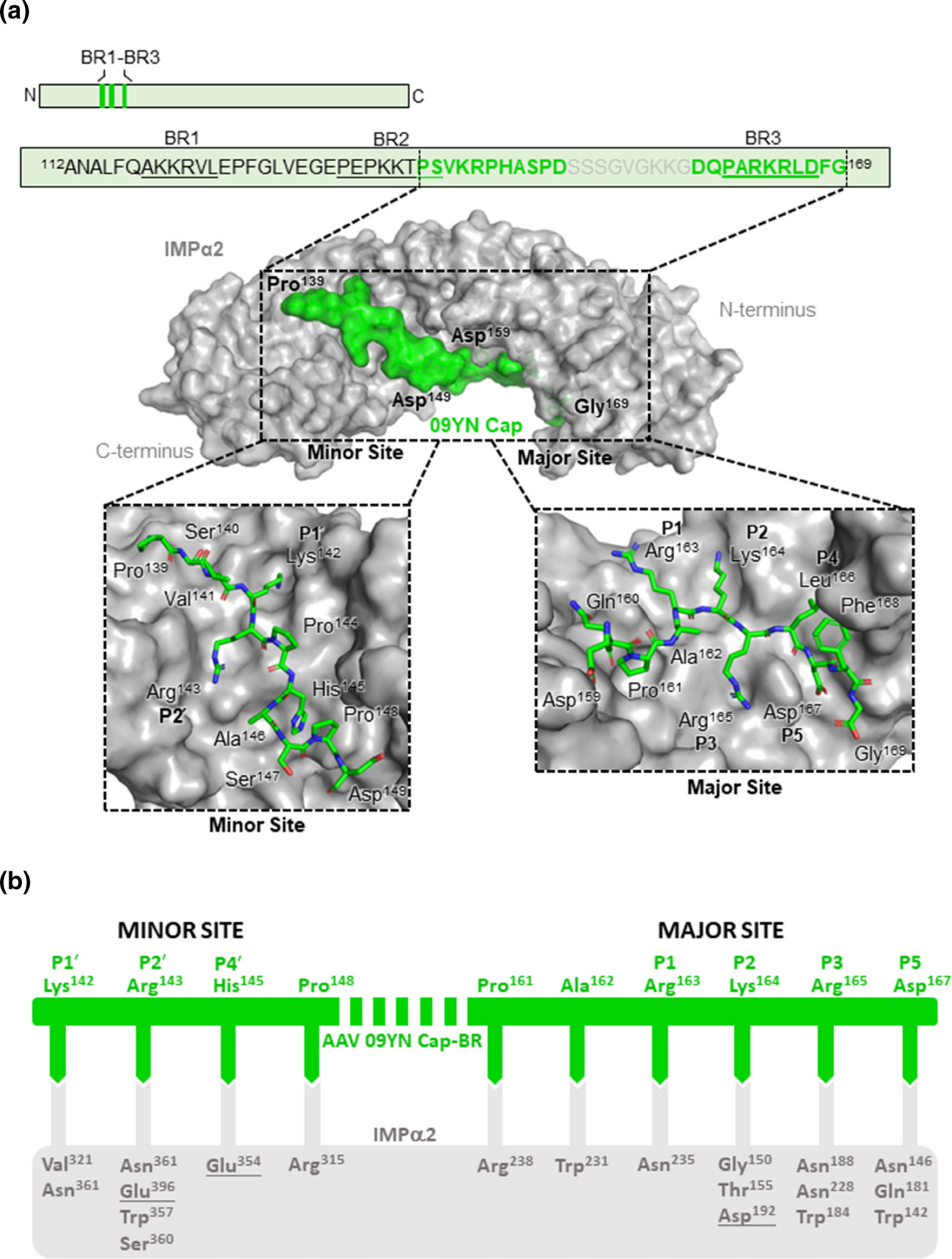
Binding affinities of importin isoforms with AAV 09YN Cap-BR. (**a**) EMSA showing AAV 09YN Cap-BR binding with all tested IMPα isoforms (spanning members from each of the three subfamilies), though this NLS does not bind with IMPβ. The AAV Cap-BR region peptide spans residues 117–167 and contains a FITC and Ahx linker (middle panel; fluorescence signal artificially coloured red for clarity). Proteins were stained using Coomassie blue stain (top panel; blue), and the overlay is represented in the bottom panel. EMSA results are representative of three independent experiments. (**b**) FP assay measuring the direct binding between AAV 09YN Cap-BR and respective importin isoforms. Strong binding in the 1–20 nM range was observed with IMPα3 (1 nM) and IMPα7 (11 nM). Moderate binding was observed with IMPα1 (483 nM) and IMPα2 (656 nM). Weaker binding was observed with IMPα5 (862 nM) and IMPβ (1136 nM). Error bars were calculated using standard deviation from the mean of three independent experiments. The error for the table was the standard error of the mean.

To determine whether AAV 09YN Cap-BR was able to bind to host nuclear import receptors (and whether any preference for individual receptor isoforms was apparent) biochemical assays were undertaken. EMSAs were performed to qualitatively observe AAV 09YN Cap-BR interactions with importin isoforms, including several IMPα family members and IMPβ. Three independent experiments were undertaken, and it was determined that AAV 09YN Cap-BR could bind with IMPα isoforms across the three subfamilies (α1, α2, α3, α5, α7) but did not bind with IMPβ (Fig. 3a). These results reveal a clear preference for AAV 09YN Cap-BR binding to IMPα. This suggests that AAV 09YN VP1 would utilize the classical nuclear import pathway, wherein the NLS binds IMPα prior to IMPα binding with IMPβ for complex formation and subsequent movement into the nucleus. This is further indicated by a lack of binding observed between 09YN Cap-BR and IMPβ demonstrating that this importin cannot act as a direct binder and that IMPα is required as an adaptor for translocation into the nucleus.

FP assays were performed to confirm EMSA results and to quantitatively measure the direct binding interactions between AAV 09YN Cap-BR and different importin isoforms (Fig. 3b). It was observed that AAV 09YN Cap-BR interacts with different importin isoforms at different magnitudes of binding strength (Fig. 3b). In particular, human IMPα3 was shown to bind very strongly at low nM concentrations (K_D_=1 nM) whilst human IMPα7 was also observed to bind strongly (K_D_=11 nM) (Fig. 3b). Human IMPα1 (K_D_=483 nM), and mouse IMPα2 (K_D_=656 nM) were shown to be much weaker binders of AAV 09YN Cap-BR compared to IMPα3 and IMPα7 but overall were determined to maintain a moderate binding affinity with AAV 09YN Cap-BR. Human IMPα5 (K_D_=862 nM) and Human IMPβ (K_D_=1136 nM) were shown to be weaker binding partners of AAV 09YN Cap-BR than IMPα1 and IMPα2. These results demonstrated that there is an appreciable difference in AAV 09YN VP1 basic regions binding with the different IMPα families, with a range of affinities from very strong binding to moderately weak. Thus, during infection of host cells, AAV 09YN capsid would likely have a strong preference for utilizing the specific isoforms IMPα3 and IMPα7 for the trafficking into the nucleus.

AAV 09YN Cap region downstream of BR2 is bound at the IMPα2 minor site and BR3 is bound at the major site as a bipartite NLS.

IMPα2 was chosen for complexing purposes as crystals of AAV 09YN Cap-BR in complex with IMPα3 were not able to be obtained of a size that yielded useable diffraction data, whilst IMPα2 crystallisation conditions are readily known. IMPα2 crystals have a high reproducibility, and diffract at high resolution for useable data. A structure was obtained of AAV 09YN Cap-BR in complex with IMPα2 in which the IMPα2 was structured into ten sequential armadillo (ARM) motifs as described previously [78] and AAV 09YN Cap-BR was bound at both the major site of IMPα2 (ARM 2-4, P1-P5 sites) and the minor site (ARM 6-8, P1ʹ-P3ʹ sites) (Fig. 2), in a manner typical of a bipartite NLS [79,81]. The overall binding buried a total surface area of 2773 Å2 and is mediated by 25 hydrogen bonds, 16 in the major binding site and nine in the minor, and three salt bridge interactions, one in the major binding site and two in the minor binding site (Fig. 2). Surprisingly, of the three basic regions, only basic region three bound to IMPα at the major site, while another region between BR2 and BR3 bound at the minor site.

Within the major site of IMPα2, AAV 09YN Cap-BR Arg^163^ interacts with the side chain of IMPα2 Asn^235^ residue at the P1 site (Fig. 2, Table S2). Lys^164^ acts as the predominant binding determinant for the specific P2 site of the major site with hydrogen bonds to Gly^150^, Thr^155^, and Asp^192^, and also maintains a salt bridge with Lys^164^ (Fig. 2, Table S2). The P3 site is occupied by AAV 09YN Cap-BR Arg^165^ which interacts with IMPα2 through hydrogen bonds formed with IMPα2 residues Asn^188^, Asn^228^ and Trp^184^. AAV 09YN Cap-BR Leu^166^ occupies the placement of the P4 binding site however no direct interactions, hydrogen bonds or salt bridges, can be observed for the residue at this site. The P5 position, however, does show AAV 09YN Cap-BR Asp^167^ forming hydrogen bonds with IMPα2 residues Asn^146^, Gln^181^ and Trp^143^ (Fig. 2, Table S2).

The solved structure also shows the minor site-binding region of AAV 09YN Cap-BR contains the canonical ‘KR’ motif typically seen. These two residues, Lys^142^ and Arg^143^ both form hydrogen bonds with the IMPα2 protein in the P1ʹ and P2ʹ sites respectively. Lys^142^ interacts through hydrogen bonds with IMPα2 Val^321^ and Asn^361^. Within the P2ʹ binding cavity, AAV 09YN Cap-BR utilizes hydrogen bonds with IMPα2 residues Asn^361^, Trp^357^, Ser^360^, and Glu^396^ (Fig. 2, Table S2), as well as forming a salt bridge with IMPα2 Glu^396^, as seen in other classical bipartite NLS structures involving IMPα2 [79,81]. The P4ʹ binding site is occupied by AAV 09YN His^145^ which holds both a hydrogen bond and salt bridge with IMPα2 residues Glu^354^. The 2.4 Å resolution model identified minor site P1ʹ, P2ʹ, P4ʹ, and major site, P1, P2, P3, and P5 binding determinants for the interaction between IMPα2 and AAV 09YN Cap-BR.

### Mutational studies confirm the importance of AAV 09YN Cap-BR residues for IMPα2 binding, however, suggest a different binding mechanism for other isoforms

**Fig. 4.**
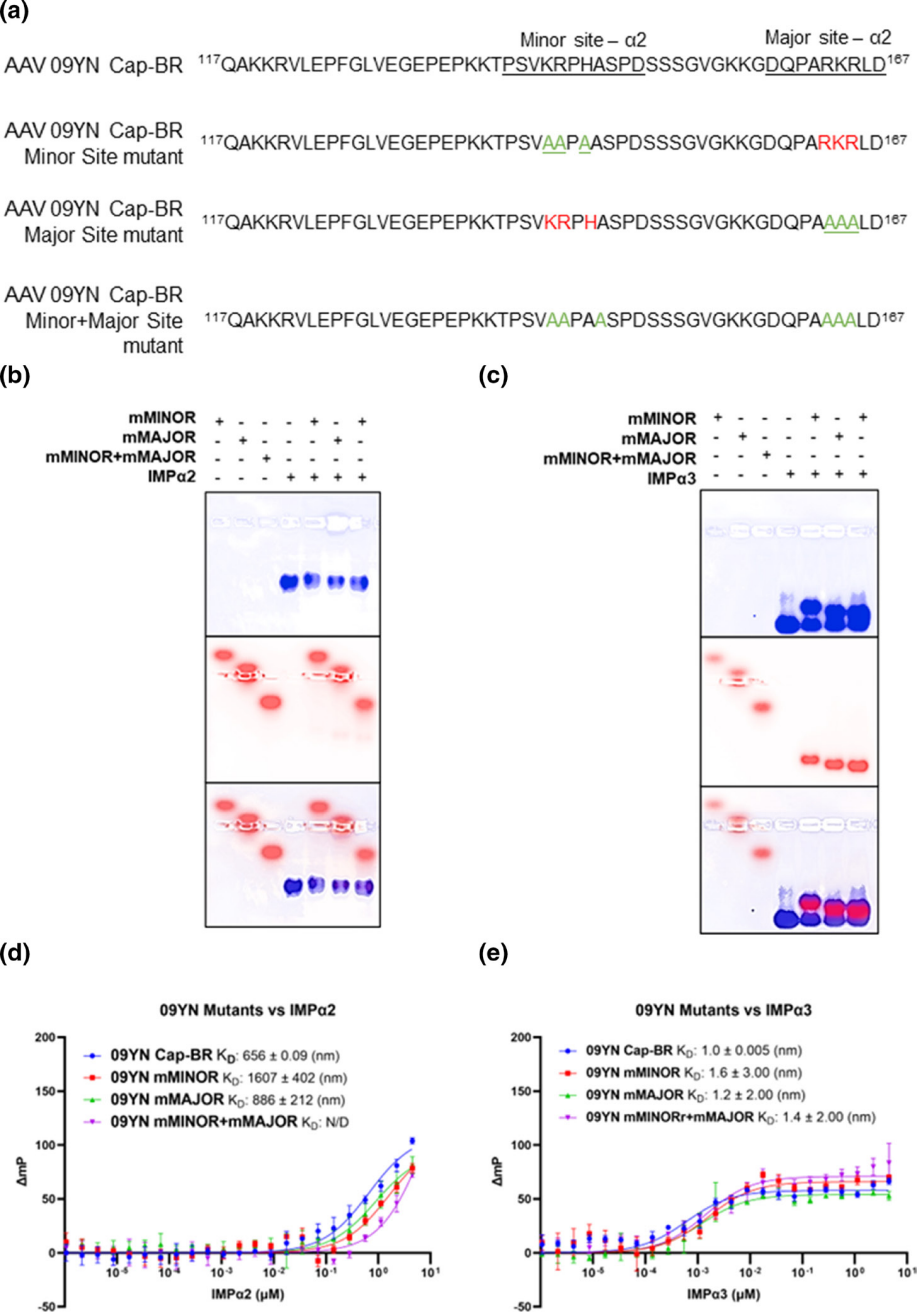
Binding affinities of AAV 09YN Cap-BR mutants during interaction with IMPα2 and IMPα3. (**a**) Sequences of non-mutated 09YN Cap-BR, and mutated 09YN Cap-BR peptides. The regions structurally determined to bind the major and minor sites of IMPα2 are underlined. For mutant sequences, green letters indicate mutated residues and red shows non-mutated residues. (**b**) EMSA demonstrates that each of the mutant AAV 09YN Cap-BR peptides are unable to bind to IMPα2. (**c**) FP assay measuring the direct binding between AAV 09YN Cap-BR and Cap-BR mutants against IMPα2. Moderate binding is observed with Cap-BR (K_D_=656 nm), and marginally weaker binding is seen with Cap-BR-mMAJOR (K_D_=886 nm). Significantly weaker binding is seen for Cap-BR-mMINOR (K_D_=1607 nm) and especially Cap-BR-mMINOR+mMAJOR which due to its low affinity a K_D_ value could not be accurately calculated. Calculated K_D_ values are displayed in the graph legend. Error bars were calculated using standard deviation from the mean of three independent experiments. The error for the K_D_ values were calculated using the standard error of the mean. (**d**) EMSA demonstrating that each of the mutant AAV 09YN Cap-BR peptides maintain their ability to bind to IMPα3. (**e**) FP assay measuring direct binding between AAV 09YN Cap-BR and Cap-BR-mutants and IMPα3 isoform. There is no significant change between non-mutated Cap-BR (K_D_=1.0 nm) and mutated AAV 09YN Cap-BR; Cap-BR-mMINOR (K_D_=1.6 nm), Cap-BR-mMAJOR (K_D_=1.2 nm) and Cap-BR-mMINOR+mMAJOR (K_D_=1.4 nm). Calculated K_D_ values are displayed in the graph legend. Error bars were calculated using standard deviation from the mean of three independent experiments. The error for the K_D_ values were calculated using the standard error of the mean. The WT AAV 09YN Cap-BR values in FP assays (panels B and D) are from the same three independent experiments performed in Fig. 3. EMSA results in panels C and E represent data from a single experiment.

Synthetic FITC labelled peptides were obtained with major and minor site mutations designed using the AAV 09YN Cap-BR:IMPα2 structure as a basis. Residues Lys^142^, Arg^143^ and His^145^ which form the P1՛, P2՛, and P4՛ positions of the minor binding site of IMPα2 were mutated to Ala residues. This mutant was labelled AAV 09YN Cap-BR-mMINOR (Fig. 4a). Residues Arg^163^, Lys^164^ and Arg^165^ of the P1, P2, and P3 positions of IMPα2 major binding site were mutated to Ala residues and the mutant labelled AAV 09YN Cap-BR-mMAJOR (Fig. 4a). A peptide with a mutated minor binding site and major binding site residues was designed and labelled AAV 09YN Cap-BR-mMINOR+mMAJOR (Fig. 4a).

FITC mutants were used in FP assays alongside IMPα2 to quantitatively determine changes in binding efficiency in a known structural context (Fig. 4d). It was observed that mutations to the minor binding site reduced the binding efficiency by almost three-fold (K_D_=1607 nm) when compared to non-mutated AAV 09YN Cap-BR. In contrast, the major binding side did not impact affinity for IMPα2 binding drastically (K_D_=886 nm), impairing binding less than two-fold. As expected, mutating both the minor and major binding site region decreased binding with IMPα2 by almost 13-fold (K_D_=8247 nm), this indicates that without these residues IMPα2 binding may not be applicable in a biological context. EMSAs were utilized to provide qualitative results of the mutant 09YN peptides against IMPα2. In this format, it was observed that the mutated 09YN peptides were not able to fully bind IMPα2 indicating that binding with IMPα2 must utilise these sites for maximum binding efficiency (Fig. 4b).

Due to AAV 09YN Cap’s high affinity for IMPα3 (Fig. 4e) these mutant peptides were tested against IMPα3 to determine the efficiency of these regions as a bipartite NLS for IMPα3. Interestingly, there was no significant change between wild-type AAV 09YN Cap-BR and mutated versions with the worst binder, Cap-BR-mMINOR, only having a 0.6× difference in the calculated K_D_ value (1.6 nm). EMSAs were utilized to provide qualitative results of the mutant 09YN peptides binding to IMPα3. It was observed that the mutated 09YN peptides can still bind IMPα3 fully (Fig. 4c). Thus, AAV 09YN Cap must utilize a different binding format when interacting with IMPα3 than it does with IMPα2.

## Discussion

Our 2.4 Å resolution model demonstrated that the AAV 09YN capsid N-terminus possesses the binding determinants typical for a bipartite NLS, with minor site P1ʹ, P2ʹ and P4ʹ regions and major site P1, P2, P3, and P5 binding determinants observed in the structural model (Fig. 2). Overall, this structural evidence suggests that not only is AAV 09YN capsid protein able to efficiently bind with a host nuclear import protein but that it would do so in a canonical manner.

The original phenotypic mutational data that defined BRs and their role in nuclear import was generated using exclusively human/primate AAV isolates [42,44, 60, 64]. This means it may be entirely possible that different isolates of AAV from non-primate animals (those not commonly explored as vectors for gene therapy) may interact with host importins via different repertoires of BRs at the N-terminus. A porcine AAV isolate was investigated regarding the structural determinants of IMPα interactions in 2021 and 2023 [43,45]. The results of these studies validate the notion of alternate BR utilization among diverse AAV isolates, with BR1 binding the minor site and a region located between BR2 and BR3 the major site forming a bipartite NLS responsible for the binding interactions with importins [45].

Though BR1 is present in the full-length VP1, it is not possessed by the shorter VP2 isoform; thus allowing for the hypothesis that BR1 may be used as part of the NLS for VP1 but not for localization of VP2. Without BR1 in VP2 and only BR2 and BR3, it is possible that only a monopartite NLS may be functional for this capsid protein isoform [45]. The possibility of porcine AAV VP1 utilizing a bipartite NLS and VP2 a monopartite may result in an overall difference in transduction efficiency between the two isoforms. Intriguingly, the Cap protein NLS of the bat AAV isolate 09YN bound IMPα2 in a different bipartite mechanism than expected from the porcine AAV data, with BR3 binding the major site of importin and a region between BR2 and BR3 interacting in the minor binding site of importin (Figs2  3). Thus, it can be hypothesised that the same region acts as a bipartite NLS for VP1 and VP2.

The structural evidence presented here provides insight into the mechanisms involved in AAV and importin interactions, and intriguingly describes a bipartite NLS utilizing regions of the N-terminal domain outside of those revealed in earlier studies [43,45]; comprising residues found in both VP1 and VP2 Cap isoforms. EMSA and FP data further solidified this evidence and showed that there is a clear difference in binding affinities of AAV 09YN capsid protein with importin isoforms (Fig. 3). Though able to bind all tested importin alpha proteins (IMPα1, IMPα2, IMPα3, IMPα5, IMPα7) in a biophysical assay (Fig. 3a), there was varying degrees of binding affinity across the isoforms and IMPβ was not bound efficiently at all (Fig. 3b). In the FP and EMSA data comparing mutations of AAV 09YN Cap-BR within the observed residues binding to the minor and major binding sites of IMPα2, it was shown that the binding efficiency of 09YN Cap to IMPα2 is affected drastically enough that these regions work as an active NLS in the context of a cell, whilst mutations had no impact on binding with IMPα3 (Fig. 4).

Collectively, these data strongly support the hypothesis that there is a preference for different importin alphas in the context of AAV 09YN cell infection and that high-affinity IMPα’s, such as IMPα3, utilize a different NLS than IMPα2. NLS specificity for IMPα isoforms is not very well categorized and is poorly understood. Considering that the relative binding of an NLS with IMPα is very similar across most structural data and that the NLS binding grooves of IMPα are highly conserved across IMPα isoforms [80,82,85] it is theorised that specificity could be directed by: the linker region of bipartite NLSs [86], the differences between the ARMs of IMPα outside of conserved binding grooves [84], or the possible overall flexibility of some IMPα isoforms such as the ‘hinge’ region of IMPα3 [83]. Without a high-resolution structural model of IMPα3 bound to AAV 09YN Cap-BR, we cannot be entirely sure how this viral capsid differs in binding between isoforms. Irrespective of questions regarding the mechanisms of IMPα specificity; our FP and EMSA data has revealed that bat AAV capsids have the potential to transduce human cells and tissues efficiently, making this virus isolate an attractive option for gene therapy. Furthermore, this evidence could be used to enhance AAV capsid engineering for targeted transduction of different cell and tissue types.

It is clear from our assessment that both porcine [43,45] and chiropteran AAV isolates, that different lineages of this viral group interact with host nuclear transport receptors through diverse repertoires of N-termini. Therefore, it is possible that of the various human/primate AAV serotypes being investigated for use in gene therapy, there may exist a spectrum of unique patterns of capsid and importin interactions. Furthermore, IMPα isoform preference may have a direct relation to transduction and subsequent transgene delivery in varying tissue and cell types. Therefore, it’s paramount that exploration of the mechanisms underlying the nuclear import of AAV is a research priority to thoroughly understand these interactions and how they can be optimized for gene therapy vector design.

## supplementary material

10.1099/jgv.0.001960 Uncited Fig. S1.

## Abbreviations

AAV: adeno-associated virus
BR: basic region
Cap: capsid
EMSA: electro-mobility shift assay
FP: fluorescence polarization
IMPα: importin alpha
IMPβ: importin beta
NLS: nuclear localization signal
VP: virion protein
