# Supplementary material for: Structural basis for nuclear import of bat adeno-associated virus capsid protein
Source: J Gen Virol. 2024 Mar 5;105(3):001960. doi: 10.1099/jgv.0.001960 (PMC10999750; doi:10.1099/jgv.0.001960)
Supplement: Uncited Fig. S1. [file jgv-105-01960-s001.pdf]

Table 1 – Data collection and refinement statistics for structure of importin-α in complex with AAV 09YN capsid basic regions 1-3

**AAV 09YN Cap-BR and IMPα2 (PDB code 8SG7)**	
**Data Collection**	
Wavelength	0.9537
Data-collection temperature (K)	298
Detector Type	DECTRIS EIGER X 16M
Detector	PIXEL
Resolution range (Å); (º)	19.95 – 2.40
Space group	P 21 21 21
Unit cell (Å)	79.06Å 90.56Å 99.82Å; 90 90 90
Total reflections	182894 (18143)
Unique reflections	32506 (3158)
Multiplicity	5.6 (5.7)
Completeness (%)	99.9 (100.00)
Mean I/σ (I)	8.8 (2.0)
Wilson B-factor Å^2	37.176
R_{pim}	0.051 (0.614)
**Refinement**	
R_{work}	0.1840 (0.2626)
R_{free}	0.2236 (0.3009)
No. of non-hydrogen atoms	3398
Macromolecules	3357
Solvent	41
Protein residues	448
Bond length r.m.s.d (Å)	0.007
Bond angle r.m.s.d (º)	0.94
Ramachandran favoured (%)	97.96
Ramachandran allowed (%)	2.04
Ramachandran outliers (%)	0.00

Table 2. Hydrogen bond and salt bridge interactions

**AAV 09YN Cap-BR hydrogen bond and salt bridge interactions with IMPα2**	
**Hydrogen bonds**	
**IMPα2**	**AAV 09YN Cap-BR**
TRP 142 [NE1]	ASP 167 [O]
ASN 146 [OD1]	ASP 167 [N]
ASN 146 [ND2]	ASP 167 [O]
GLY 150 [O]	LYS 164 [NZ]
THR 155 [OG1]	LYS 164 [NZ]
GLN 181 [NE2]	ASP 167 [OD2]
TRP 184 [NE1]	ARG 165 [O]
ASN 188 [OD1]	ARG 165 [N]
ASN 188 [ND2]	ARG 165 [O]
ASP 192 [OD1]	LYS 164 [NZ]
ASN 228 [OD1]	ARG 165 [NH1]
ASN 228 [OD1]	ARG 165 [NH2]
TRP 231 [NE1]	ALA 162 [O]
ASN 235 [ND2]	ARG 163 [O]
ARG 238 [NH1]	PRO 161 [O]
ARG 238 [NH2]	PRO 161 [O]
ARG 315 [NH1]	PRO 148 [O]
VAL 321 [O]	LYS 142 [NZ]
GLU 354 [OE2]	HIS 145 [NE2]
TRP 357 [NE1]	ARG 143 [O]
SER 360 [OG]	ARG 143 [NH1]
ASN 361 [O]	LYS 142 [NZ]
ASN 361 [OD1]	ARG 143 [N]
ASN 361 [ND2]	ARG 143 [O]
GLU 396 [OE1]	ARG 143 [NH1]
**Salt Bridges**	
**IMPα2**	**AAV 09YN Cap-BR**
ASP 192 [OD1]	LYS 164 [NZ]
GLU 354 [OE2]	HIS 145 [NE2]
GLU 396 [OE1]	ARG 143 [NH1]

Supplementary Figure 1

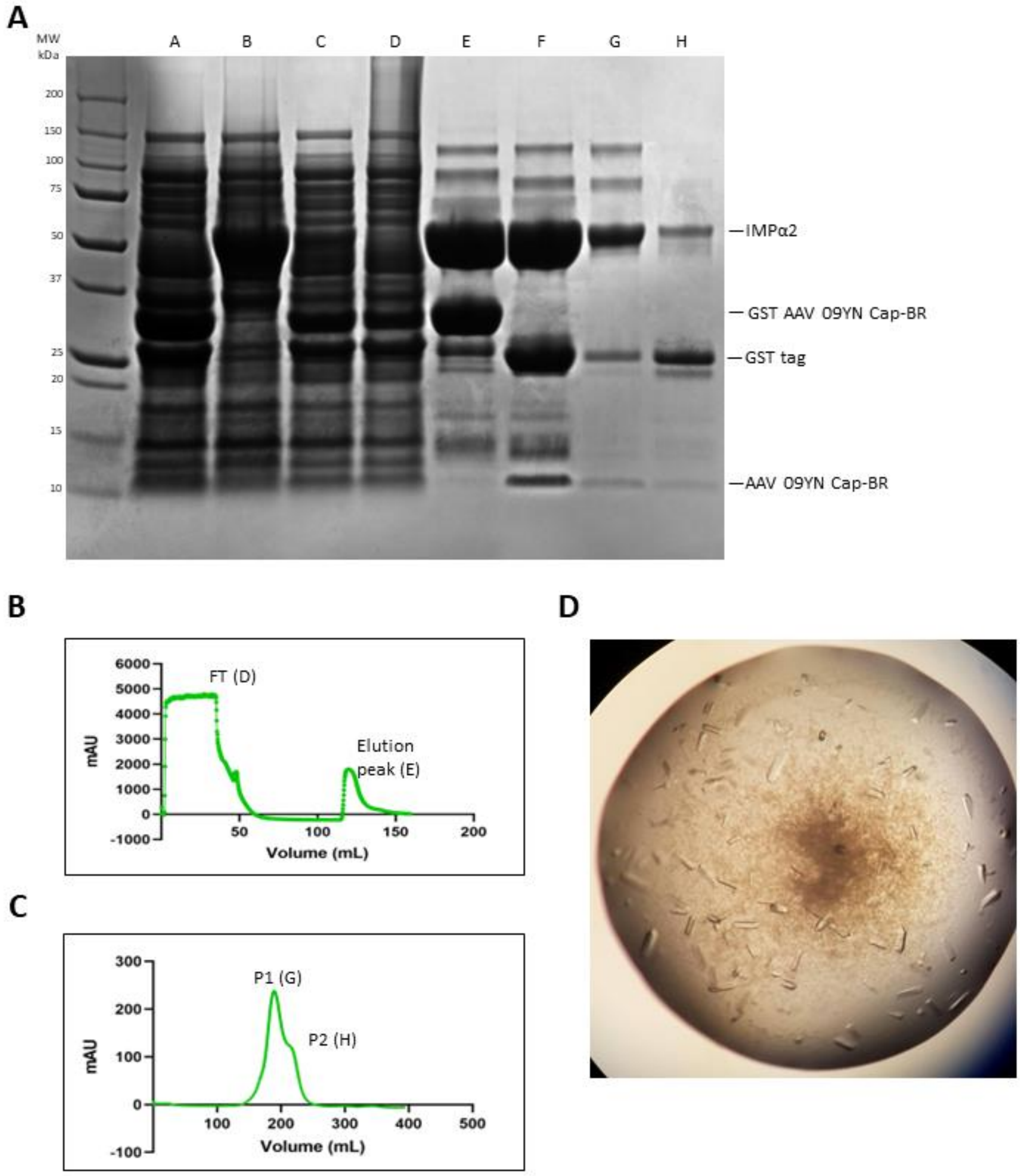

Figure 1. Purification of GST-AAV 09YN Cap-CR fusion in complex with IMPα2. (A) SDS-PAGE analysis of the purification process. Lane A, whole cell lysate of cells expressing GST-AAV 09YN Cap-BR. Lane B, whole cell lysate of *E*. *coli* expressing recombinant mouse His-IMPα2. Lane C, soluble extract of the combined whole cell lysates. Lane D, flow through fraction of the Ni-affinity column. Lane E, Ni-affinity column elution of the His-IMPα2:GST-AAV 09YN Cap-BR complex. Lane F, Ni-affinity purified His-IMPα2:GST-AAV 09YN Cap-BR complex digested with TEV protease. Lane G, elution fraction P1 from size-exclusion chromatography. Lane H, elution fraction P2 from size-exclusion chromatography. **(B)** UV trace of Ni-affinity co-purification of mouse His-IMPα2 with GST-AAV 09YN Cap-BR. **(C)** UV trace of size-exclusion chromatography of the TEV-cleaved His-IMPα2:AAV 09YN Cap-BR complex. **(D)** Protein crystals of IMPα2 in complex with AAV 09YN Cap-BR. Rod-shaped crystals formed after 2 days of incubation at room temperature with 0.75 M Na citrate, 0.1 M HEPES pH 7.5.
